# Effects of AMF on plant nutrition and growth depend on substrate gravel content and patchiness in the karst species *Bidens pilosa* L

**DOI:** 10.3389/fpls.2022.968719

**Published:** 2022-09-29

**Authors:** Kaiping Shen, Yuejun He, Xinyang Xu, Muhammad Umer, Xiao Liu, Tingting Xia, Yun Guo, Bangli Wu, Han Xu, Lipeng Zang, Lu Gao, Min Jiao, Xionggui Yang, Jiawei Yan

**Affiliations:** ^1^ Forestry College, Research Center of Forest Ecology, Guizhou University, Guiyang, China; ^2^ Forestry Survey and Planning Institute of Guizhou Province, Guiyang, China; ^3^ College of Eco-Environmental Engineering, Guizhou Minzu University, Guiyang, China

**Keywords:** arbuscular mycorrhizae, gravel, heterogeneity, karst, patch, substrate

## Abstract

Karst ecosystems represent a typical heterogeneous habitat, and it is ubiquitous with varying interactive patches of rock and soil associated with differential weathering patterns of carbonate rocks. Arbuscular mycorrhizae fungi (AMF) play an important role in regulating plant growth and nutrition in heterogeneous karst habitats. However, it remains unclear how AMF affects the growth and nutrition of plants in heterogeneous karst soil with varying patches and weathering gravel. A heterogeneous experiment with *Bidens pilosa* L. was conducted in a grid microcosm through patching karst soil with different gravel contents. The experimental treatments included the AMF treatments inoculated with (M^+^) or without (M^-^) fungus *Glomus etunicatum*; the substrate patchiness treatments involved different sizes of the homogeneous patch (Homo), the heterogeneous large patch (Hetl), and the heterogeneous small patch (Hets); the substrate gravel treatments in the inner patch involved the free gravel (FG), the low gravel (LG) 20% in 80% soil, and the high gravel (HG) 40% in 60% soil. Plant traits related to growth and nutrients were analyzed by comparing substrate gravel content and patch size. The results showed that AMF was more beneficial in increasing the aboveground biomass of *B. pilosa* under the LG and HG substrates with a higher root mycorrhizal colonization rate than under the FG substrate with a lower root mycorrhizal colonization rate. AMF enhanced higher growth and nutrients for *B. pilosa* under the LG and HG substrates than under the FG substrate and under the Hets than under the Hetl. Moreover, AMF alleviated the limited supply of N for *B. pilosa* under all heterogeneous treatments. Furthermore, the response ratio LnRR of *B. pilosa* presented that the substrate gravel promoted the highest growth, N and P absorption than the substrate patchiness with M^+^ treatment, and the gravel content had a more effect on plant growth and nutrition as compared to the patch size. Overall, this study suggests that plant growth and nutrition regulated by AMF mainly depend on the substrate gravel content rather than the spatial patchiness in the heterogeneous karst habitat.

## Introduction

Karst landscapes developed from carbonate rock present a high spatial heterogeneity in habitats (Zhang et al., 2018), and due to weathering of carbonate rock, these rocks constantly dissolve, forming many gravel particles and micro-landscapes such as rock surfaces, gullies, pits, and crevices on a small scale. It leads to a discontinuous distribution of soil surface cover and has a huge difference in soil thickness, resulting in both high soil substrate and spatial heterogeneity in karst habitats (Liu et al., 2010; Zhou et al., 2010). Therefore, the habitat heterogeneity of the karst ecosystem is induced by the variable spatial and substrate composed of different gravel content as the essential features of the karst habitat (Cao et al., 2003; Zhang et al., 2007), which exacerbates the imbalance of crucial resources required to plants (Kotliar and Wiens, 1990; Wijesinghe and Hutchings, 1997). It is a critical component of spatial heterogeneity, and the spatial scale may significantly affect the growth of individual plant species (Kotliar and Wiens, 1990). Meanwhile, differences in substrate composition can affect plant growth by altering the belowground activity of plants (Hutchings et al., 2003). Therefore, the patchiness and gravel of karst soil substrate might differentially influence plant growth and nutrient acquisition (Facelli and Facelli, 2002; Maestre et al., 2005). In addition, karst ecosystems with high spatial heterogeneity hold affluent microbial diversity (Peng et al., 2019), especially some functional microorganisms such as arbuscular mycorrhizae fungi (AMF) (Wei et al., 2011; Lin et al., 2019), which involved in enhancement of plant growth and nutrient utilization (He et al., 2019; Shen et al., 2020; Guo et al., 2021) in karst habitats. Therefore, mycorrhizal symbiosis may play an essential role in heterogeneous karst habitats.

AMF is a class of soil microbes that can form symbiotic relationships with more than two-thirds of terrestrial plants, benefiting plants by deploying dense mycelium in the soil to access mineral nutrients (Smith and Read, 2008). In response, the host plant provides a carbon source for the AMF to support mycelium growth (Selosse et al., 2006). Spatial heterogeneity affects plant growth and nutrient availability, and AMF may strongly modify the effects of heterogeneity on the growth and nutrient acquisition of plants (Facelli and Facelli, 2002). Previous research shows that plants growing in heterogeneous soil environments depend more on AMF to obtain nutrients (Liang et al., 2022). Additionally, nitrogen (N) and phosphorus (P) are essential nutrients during plant growth and development (Wright et al., 2011). The AMF colonized on the host plant’s root system can effectively enhance N and P nutrient uptake (Govindarajulu et al., 2005; Adesemoye et al., 2008), especially in limited N and P availability in the degraded karst ecosystem.

Plant biomass generally characterizes the adaptive ability of a plant to the external environment, which is influenced by the habitat (Qi et al., 2019). The root and shoot biomass allocation ratio (R/S ratio) is an important trait to describe plant adaptation to drought stress or other environmental stresses (Hutchings and John, 2004; Bonifas and Lindquist, 2006). Notably, the optimal partitioning theory (OPT) states that plants preferentially increase the biomass of organs that have access to more limited resources for growth (Gedroc and Coleman, 1996; McCarthy and Enquist, 2007). Therefore, according to the OPT theory, high soil nutrient availability enables plants to enhance the aboveground part’s biomass; conversely, low soil nutrient availability enhances the belowground part’s biomass. Meanwhile, plants often benefit more from a symbiotic relationship with AMF in soil nutrient-scarce environments than in soil nutrient-rich environments (Hoeksema et al., 2010; Zaller et al., 2011). However, it has not clarified whether the biomass partition of plants still follows the OPT when plants are combined with AMF in heterogeneous karst areas.

Plant-fungi symbioses work in heterogeneous spaces in natural environments (Ayesu and Gyabaah, 2014). Generally, soils are heterogeneous in natural habitats (Xue et al., 2016; Cao et al., 2022), and soil substrate composition tends to have large variations within a small distance (Wijesinghe et al., 2005), which likely influences plant growth and plant-fungal interactions. Additionally, plant growth responses to heterogeneous resources may also change with heterogeneous patches (van der Waal et al., 2011). However, how AMF regulates plant growth and nutrient utilization under patchy habitats with varying soil compositions in the karst ecosystem remains unclear. Given that AMF can enhance host plants’ growth and nutrition (He et al., 2019), especially under low nutrient conditions (Adesemoye et al., 2008). Meanwhile, a large amount of gravel might reduce nutrient availability (Fu et al., 2022). In addition, Wijesinghe and Hutchings (1997) and Wang et al. (2016) concluded that the plant under large heterogeneous patches produced larger biomass than under small heterogeneous patches. Therefore, we hypothesized that AMF enhances karst plant growth and nutrition more in the soil substrate mixed with gravel than in the free gravel substrate in the patchy habitat (H1), and AMF enhances karst plant growth and nutrition more in the large patch than in the small patch (H2). Further, according to Wijesinghe and Hutchings (1997), the resource acquisition capacity of plants depends on the patch size of heterogeneity. Therefore, we hypothesized that karst plant growth and nutrient acquisition depend on spatial patchiness rather than gravel substrate (H3). Thus, a heterogenous experiment was conducted regarding AMF inoculating with karst plants in mosaic substrate patches of different gravel contents to explore the plant-maintaining mechanisms in heterogeneous karst habitats.

## Materials and methods

### Experimental design

**Figure 1:**
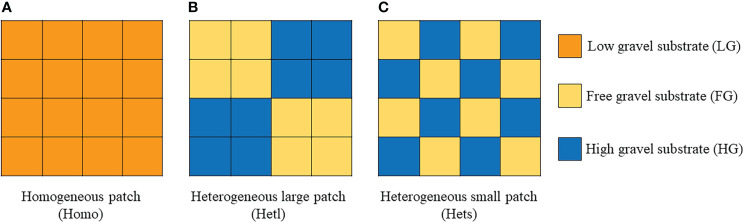
Schematic diagram of the experimental design. The experiment comprises three factors. The first factor involved the AMF treatments, including inoculation with (M^+^) or without (M^-^) fungus. The second factor involved the substrate patchiness treatments, including the homogeneous patch (Homo) **(A)**, the heterogeneous large patch (Hetl) **(B)** and the heterogeneous small patch (Hets) **(C)**. The third factor involved the substrate gravel treatments, including the free gravel (FG) substrate, the low gravel (LG) substrate of 20% gravel, and the high gravel (HG) substrate of 40% gravel. In particular, the growth substrate remained uniform in mass and volume in any treatment except for patch size.

An experiment was performed with a square microcosm (26 cm × 26 cm × 15 cm, caliber × bottom diameter × height) made up of polypropylene plastic. The microcosm was divided into 16 small grid cells through a movable grid plate for spatially forming a heterogeneous patch by filling with growth substrates quantitatively in each grid (**Figure 1**), which involved full factor experiments of AMF, substrate patchiness, and substrate gravel. The AMF treatments were inoculated with (M^+^) or without (M^-^) fungus. The substrate patchiness treatments involved the homogeneous patch (Homo), the heterogeneous large patch (Hetl), and the heterogeneous small patch (Hets). The substrate gravel was treated by three different gravel contents in the inner patches, including the free gravel substrate with 100% soil (FG), the low gravel substrate with a mixture of 80% soil and 20% gravel (LG), and the high gravel substrate with a mixture of 60% soil and 40% gravel (HG) (**Figure 1**). Especially, the growth substrate remained uniform in mass and volume in any treatment except for patch size. For the M^+^ treatment, we added 50 g of inoculum to each device; for the M^-^ treatment, an equal amount of sterilized inoculum was added to each device.

Additionally, 10 ml of filtrate taken from inoculum was added to M^-^ treatments to make the M^+^ and M^-^ treatments have the same microbiota except for the target fungus. Finally, five seeds were sown into each grid. One plant was kept in each cell after seed germination, and 16 seedlings remained per microcosm ultimately. There were eight replicates involving 48 microcosms, including 768 plants through two AMF levels (M^+^ vs. M^-^), three substrate patchiness levels (Homo vs. Hetl vs. Hets), along with the substrate gravel in the inner patch through three gravel content levels (FG vs. LG vs. HG). After 12 weeks of culture in a greenhouse on the western campus of Guizhou University (106˚220 E, 29˚490 N, 1120 m above sea level), all plant and soil materials were harvested for measurement.

### Plant material

*Bidens pilosa* L. is an annual herb of the Asteraceae and is widely distributed in the karst areas of southwest China (Li et al., 2021). *B. pilosa* is the typical pioneer succession species of karst vegetation documented by our field surveys, and our previous found that it has a high rate of mycorrhizal colonization (He et al., 2019; Han et al., 2020; Li et al., 2022; Sun et al., 2022). Therefore, *B. pilosa* was selected as the plant material in this study. Plant seeds were collected from a typical karst habitat in Huaxi District, Guiyang City, Guizhou province, China. The seeds were stored in a freezer at 4°C before use. In addition, all seeds were surface-sterilized using KMnO_4_ of 0.1% for 10 minutes, subsequently rinsed three times in sterile deionized water to avoid contamination, and then soaked in the water for 12 hours to promote germination.

### Plant growing substrate

The growing substrates were composed of a different proportion of soil and gravel collected from a typical karst habitat in Huaxi District, Guiyang City, Guizhou province, China. The soil was air-dried naturally after removing remnants of litter and roots. In addition, all gravel was sieved through a 4 mm mesh to ensure uniform gravel size. The soil and gravel were sterilized at 0.14 Mpa at 126°C for 1 hour separately. The soil had total nitrogen (TN) of 622 mg·kg^-1^, available nitrogen (AN) of 0.315 mg·kg^-1^, total phosphorus (TP) of 127 mg·kg^-1^, available phosphorus (AP) of 0.163 mg·kg^-1^, total potassium (TK) of 378 mg·kg^-1^, available potassium (AK) of 532.183 mg·kg^-1^, and the measurement method refers to Bao (2000).

### AM fungus propagation

The AM fungus *Glomus etunicatum* was purchased from the Institute of Nutrition Resources, Beijing Academy of Agricultural and Forestry Sciences, BGA0046 and propagated for treatments. It was propagated with *Trifolium repens* growth in sterilized limestone soil substrate for 4 months and then harvested naturally air-dried soil after removing the *T. repens*, stored at 4°C until use. Additionally, the AM fungus inoculum included approximately 150 spores per 10 g of soil, hyphal piece, and colonized root segments.

### Measurements and calculations

The grid line-intersect method determined the root mycorrhizal colonization rate (Giovannetti and Mosse, 1980). We used a 100 cm ruler to measure the seedling height and a standard vernier caliper to measure the ground diameter. The biomass was determined by weighing individual materials after drying at 75°C for 48 h. The R/S ratio was the root-to-shoot biomass ratio. The diffusion method with the semi-micro open method and the molybdenum antimony anti-colorimetric method were adopted for N and P concentrations, respectively (Bao, 2000). The N and P accumulations were the nutrient concentrations of each plant multiplied by the biomass of each plant. The N/P ratio was the ratio of the N to P accumulation ratio.

### Calculation of effect size

In order to measure the effect size of substrate patchiness and substrate gravel, the response ratio (LnRR) was calculated by log-transforming trait values of seedling height, ground diameter, biomass, R/S ratio, N and P accumulation (Hedges et al., 1999). The effect size of substrate patchiness was obtained by comparing trait values of the homogeneous patch to heterogeneous large or small patches. The effect size of substrate gravel was obtained by comparing trait values of the free gravel to the low or high gravel content. Thus, the modified approach was applied based on Hedges et al. (1999) and Wang et al. (2016) as follows:


. $$\text{LnRR}=\text{ln}\left(X_{c}/X_{t}\right)$$

The *X_c_* is the mean of the trait values under the homogeneous patch or the free gravel substrate across the 16 replicates, and the *X_t_* is the trait values under the large patch and small patch or the low and high gravel content in each replicate. The positive value (LnRR > 0) indicates negative effects, and the negative value (LnRR< 0) indicates positive effects (Hedges et al., 1999).

### Statistical analysis

All statistical analyses were performed with SPSS (26, USA, 64 Bit) software. All data were tested for normality and homogeneity of variance before analysis. Three-way ANOVA were used to test the effects of AMF (M^+^ vs. M^-^), substrate patchiness (Homo vs. Hetl vs. Hets) and substrate gravel (LG vs. FG vs. HG) and their interactions on the seedling height, ground diameter, biomass, R/S ratio, N accumulation, P accumulation and N/P ratio. Significant differences between M^+^ and M^-^, among Homo, Hetl and Hets, and among LG, FG and HG on root mycorrhizal colonization rate, seedling height, ground diameter, biomass, R/S ratio, N accumulation, P accumulation, N/P ratio and the response ratio LnRR at 0.05 level were determined with the least significant difference (LSD) test. Additionally, two-way ANOVA were used to test the effects of AMF (M^+^ vs. M^-^) and patch size (Hetl vs. Hets), or AMF (M^+^ vs. M^-^) and gravel content (LG vs. HG) and the interactions on the LnRR of seedling height, ground diameter, biomass, R/S ratio, N accumulation, P accumulation. All graphics were generated with Origin 2018 (95C, USA, 64 Bit) software.

## Results

### The root mycorrhizal colonization rate of *B. pilosa*

The root mycorrhizal colonization rate under Hets with GH substrate was significantly higher than under other treatments (**Table 1**). The root mycorrhizal colonization rate under the heterogeneous small patch was significantly higher than under the heterogeneous large patch, and under the high gravel and low gravel substrates was significantly higher than under the free gravel substrate.

**Table 1:** The root mycorrhizal colonization rate of *B. pilosa* under the inoculated treatments.

Treatments		Mycorrhizal colonization rate(%)
Homo	LG	58.75 ± 0.66 bc
Hetl	FG	48.13 ± 0.97 d
	HG	60.67 ± 0.59 b
Hets	FG	58.21 ± 0.67 c
	HG	63.79 ± 0.49 a

The different lowercase letters (a, b, c, d) indicate significant differences among various heterogeneous treatments (*P* < 0.05).

### The seedling height, ground diameter, biomass and R/S ratio of *B. pilosa*

**Figure 2:**
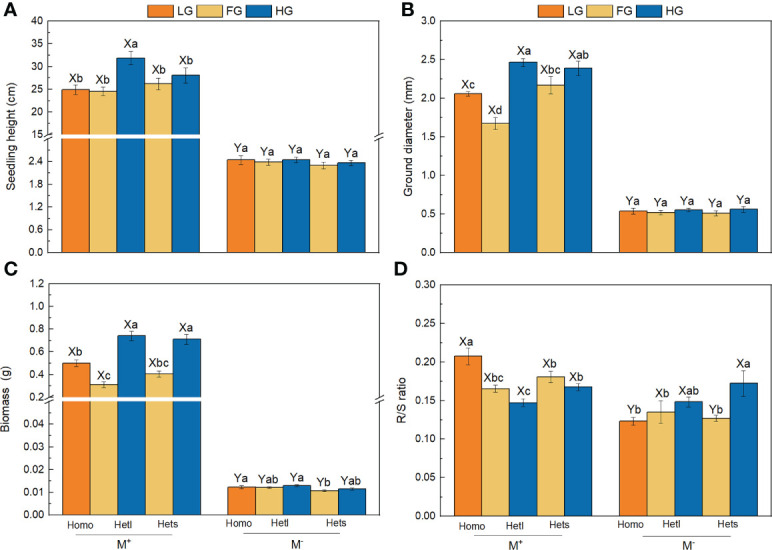
The seedling height, ground diameter, biomass and R/S ratio of *B pilosa*. For the seedling height **(A)**, ground diameter **(B)**, biomass **(C)**, and R/S ratio **(D)** of B. pilosa, M^+^ = with AMF; M^–^ = without AMF; Homo = homogeneous patch; Hetl = heterogeneous large patch; Hets = heterogeneous small patch. LG = low gravel substrate; FG = free gravel substrate; HG = high gravel substrate. Different capital letters (X, Y) above the bars indicate significant differences between M^+^ and M^-^ treatments; different lowercase letters (a–d) above the bars indicate significant differences among various heterogeneous treatments (*P* < 0.05).

The AMF treatments significantly affected the seedling height, ground diameter, biomass and R/S ratio (**Table 2**). AMF significantly enhanced seedling height, ground diameter and biomass of *B. pilosa* under all treatments (**Figures 2A–C**) and significantly improved the R/S ratio under the Homo treatment with LG substrate and Hets treatment with FG substrate (**Figure 2D**). The substrate patchiness treatments significantly affected the seedling height and ground diameter, and had non-significant effect on the biomass or the R/S ratio (**Table 2**). With M^+^ treatment, the seedling height, ground diameter and biomass under Hetl were higher than under Hets in HG substrate, and under Hets were higher than under Hetl in FG substrate; under M^-^ treatment, there was no significant difference among various treatments (**Figures 2A–C**). In addition, the R/S ratio in Homo was significantly higher than in Hetl and Hets with M^+^ treatment, and the R/S ratio in Homo was lower than in Hetl and Hets with M^-^ treatment (**Figure 2D**). The substrate gravel treatments significantly affected the seedling height, ground diameter and biomass, and had no significant effect on the R/S ratio (**Table 2**). Under M^+^ treatment, the seedling height, ground diameter and biomass in HG substrate were higher than in LG and FG substrates; under M^-^ treatment, there was no significant difference among various treatments for seedling height and ground diameter (**Figures 2A–C**). In addition, under M^+^ treatment, the R/S ratio in LG substrate was significantly higher than in FG and HG substrates, and in LG substrate was lower than in FG and HG substrates under M^-^ treatment (**Figure 2D**). The interaction of AM × SG significantly influenced the seedling height, ground diameter, biomass. and R/S ratio (**Table 2**). Overall, AMF significantly enhanced the seedling height, ground diameter and biomass of *B. pilosa*. It also promoted higher growth under the substrate mixed with gravel than under free gravel substrate as well as under heterogeneous small and heterogeneous large patches with high gravel substrate than under homogeneous patch.

**Table 2:** The three-way ANOVA for the effects of AMF (M^+^ vs. M^-^), substrate patchiness (Homo vs. Hetl vs. Hets) and substrate gravel (LG vs. FG vs. HG) treatments on the seedling height, ground diameter, and R/S ratio of *B. pilosa*.

Factors	df	Seedling height		Ground diameter		Biomass		R/S ratio	
		*F*	*P*	*F*	*P*	*F*	*P*	*F*	*P*
AMF (AM)	1	2117.254	**< 0.001**	1783.397	**< 0.001**	1306.526	**< 0.001**	44.245	**< 0.001**
Substrate patchiness (SP)	2	0.785	0.378	4.970	**< 0.05**	0.823	0.367	3.284	0.073
Substrate gravel (SG)	2	12.863	**< 0.01**	33.969	**< 0.001**	111.113	**< 0.001**	0.921	0.340
AM × SP	2	0.574	0.451	5.030	**< 0.05**	0.970	0.327	0.485	0.488
AM × SG	2	12.186	**< 0.01**	24.369	**< 0.001**	110.126	**< 0.001**	10.187	**< 0.01**
SP × SG	4	4.502	**< 0.05**	8.856	**< 0.01**	3.175	0.078	1.702	0.195
AM × SP × SG	4	4.515	**< 0.05**	9.983	**< 0.01**	3.185	0.078	0.906	0.344

The “< 0.05” indicates a significant effect, the “< 0.01” and “< 0.001” indicate an extremely significant effect.

### The N accumulation, P accumulation and N/P ratio of *B. pilosa*

**Figure 3:**
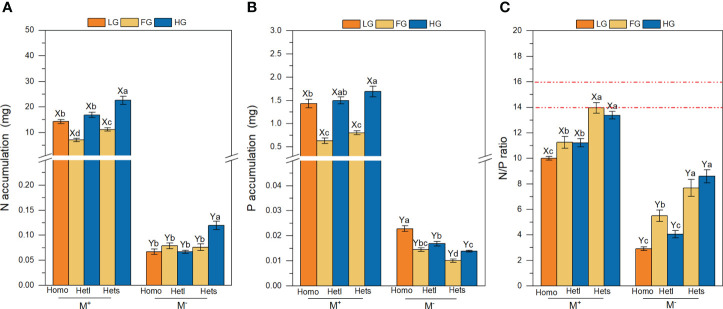
The N accumulation, P accumulation and N/P ratio of *B pilosa*. For the N accumulation **(A)**, P accumulation **(B)**, and N/P ratio **(C)** of B. pilosa, M^+^ and M^–^; Homo, Hetl and Hets; LG, FG and HG substrates, implications are the same as in **Figure 2**. The implications of the X and Y, the (a–d) above the bars are the same as in **Figure 2**.

The AMF treatments significantly affected the N accumulation, P accumulation and N/P ratio of *B. pilosa* (**Table 3**). AMF significantly improved the N accumulation, P accumulation and N/P ratio under all treatments (**Figures 3A–C**). The substrate patchiness treatments significantly affected the N accumulation, P accumulation and N/P ratio of *B. pilosa* (**Table 3**). With the M^+^ treatment, the N and P accumulation under Homo was significantly higher than under Hets and Hetl with FG substrates, and under Hets were higher than under Hetl with HG and FG substrates (**Figures 3A, B**). With the M^-^ treatment, the N accumulation under Hets with HG substrate was significantly higher than other patches, and the P accumulation exhibited that Homo > Hetl > Hets (**Figures 3A, B**). In addition, the N/P ratio exhibited that Homo< Hetl< Hets under M^+^ and M^-^ treatments (**Figure 3C**). The substrate gravel treatments significantly affected the N and P accumulation and had a non-significant effect on the N/P ratio (**Table 3**). Specifically, the N and P accumulation with M^+^ treatment exhibited HG > LG > FG; under M^-^ treatment, the P accumulation exhibited LG > HG > GF (**Figures 3A, B**); the N/P ratio in LG substrate was significantly lower than in FG and HG substrates under M^+^ and M^-^ treatments (**Figure 3C**). The interaction of AM × PH and AM × SG significantly influenced the N and P accumulation, the interaction of AM × SP × SG significantly influenced the N/P ratio (**Table 3**). Overall, AMF significantly increased the N accumulation, P accumulation and N/P ratio of *B. pilosa*, and AMF stimulated greater nutrition under the substrate mixed with gravel than under the free gravel substrate as well as under heterogeneous small and heterogeneous large patches with high gravel substrate than under homogeneous patch.

**Table 3:** The three-way ANOVA for the effects of AMF (M^+^ vs. M^-^), substrate patchiness (Homo vs. Hetl vs. Hets) and substrate gravel (LG vs. FG vs. HG) treatments on the N accumulation, P accumulation, and N/P ratio of *B. pilosa*.

Factors	df	N accumulation		P accumulation		N/P ratio	
		*F*	*P*	*F*	*P*	*F*	*P*
AMF (AM)	1	1134.669	**< 0.001**	1173.590	**< 0.001**	814.480	**< 0.001**
Substrate patchiness (SP)	2	22.717	**< 0.001**	5.158	**< 0.05**	88.120	**< 0.001**
Substrate gravel (SG)	2	104.364	**< 0.001**	126.237	**< 0.001**	0.848	0.360
AM × SP	2	22.264	**< 0.001**	5.594	**< 0.05**	2.240	0.138
AM × SG	2	103.744	**< 0.001**	124.500	**< 0.001**	0.003	0.960
SP × SG	4	0.651	0.422	0.013	0.911	2.210	0.141
AM × SP × SG	4	0.568	0.453	0.009	0.925	5.553	**< 0.05**

The “< 0.05” indicates a significant effect, the “< 0.01” and “< 0.001” indicate an extremely significant effect.

### The LnRR of plant growth and nutrition across patch size and gravel content

**Figure 4:**
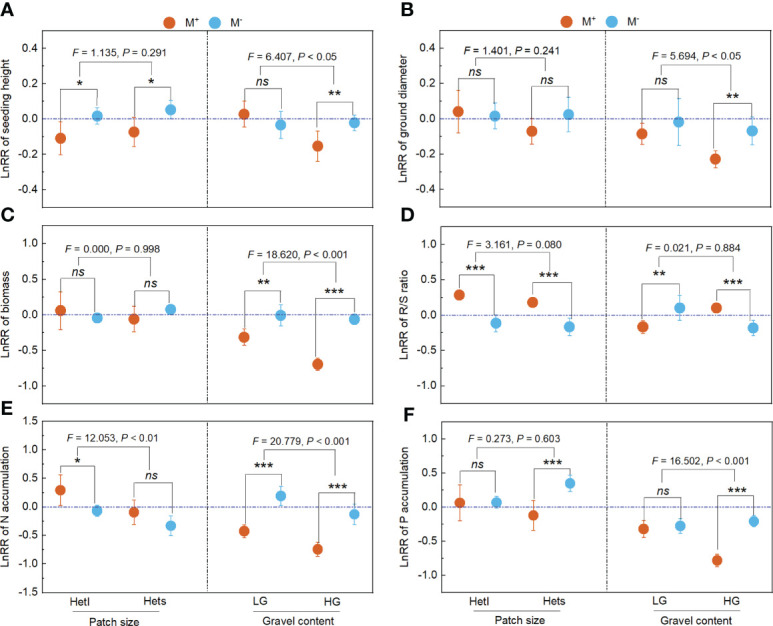
The LnRR of plant growth and nutrition across patch size and gravel content. M^+^ and M^–^; Hetl and Hets; LG and HG, implications are the same as in **Figure 2**. the * indicate significant differences between M^+^ and M^–^ (* indicate *P*< 0.05; ** indicate *P*< 0.01; *** indicate *P*< 0.001), the ns indicates non-significant differences between M^+^ and M^–^. Additionally, the *F* and *P* values were obtained from the two-way ANOVA, indicating the effect of patch size and gravel content on the LnRR for the seedling height **(A)**, ground diameter **(B)**, biomass **(C)**, R/S ratio **(D)**, N accumulation **(E)**, and P accumulation **(F)** of *B*. *pilosa*.

For patch size, with M^-^ treatment, the Hets showed negative effects on seedling height, ground diameter, biomass, R/S ratio and P accumulation of *B. pilosa*; with M^+^ treatment, the Hets showed positive effects on seedling height, ground diameter, biomass, R/S ratio, N accumulation and P accumulation of *B. pilosa* (**Figures 4A–F**). For gravel content, AMF significantly improved the positive effects on seedling height, ground diameter, biomass, N accumulation and P accumulation of *B. pilosa* under HG conditions (**Figures 4A–C, E, F**). Further, the gravel content significantly influenced the LnRR of the seedling height, ground diameter, biomass, N accumulation and P accumulation (**Figures 4A–C, E, F**), and the patch size non-significantly influenced the LnRR of the seedling height, ground diameter, biomass and P accumulation (**Figures 4A–C, F**). Overall, plant growth and N and P accumulation regulated by AMF depends mainly on the gravel content rather than the patch size.

### The LnRR of plant growth and nutrition across substrate patchiness and substrate gravel

**Figure 5:**
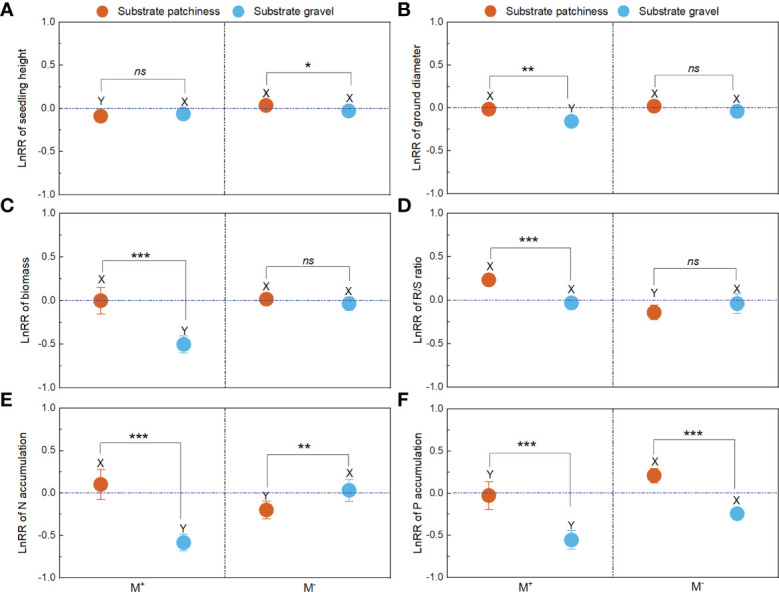
The LnRR of plant growth and nutrition across substrate patchiness and substrate gravel. For LnRR of the seedling height **(A)**, ground diameter **(B)**, biomass **(C)**, R/S ratio **(D)**, N accumulation **(E)**, and P accumulation **(F)**, M^+^ and M^–^ are the same as in **Figure 2**. The different capital letters (X, Y) indicate significant differences between M^+^ and M^-^ treatments; the * indicate significant differences between substrate patchiness treatments and substrate gravel treatments (* indicate *P*< 0.05; ** indicate *P* < 0.01; *** indicate *P*< 0.001) and the ns indicates non-significant differences between substrate patchiness treatments and substrate gravel treatments.

AMF significantly increased the positive effects on seedling height and P accumulation under substrate patchiness (**Figures 5A, F**). AMF significantly increased the positive effects on ground diameter, biomass, N accumulation and P accumulation under substrate gravel (**Figures 5B, C, E, F**). Comparing substrate patchiness and substrate gravel, with M^+^ treatment, the LnRR of ground diameter, biomass, N accumulation and P accumulation in substrate gravel was significantly higher than in substrate patchiness (**Figures 5B, C, E, F**), and the LnRR of R/S ratio in substrate gravel was significantly lower than in substrate patchiness (**Figure 5D**).

## Discussion

### Mycorrhizal efficiencies on growth and nutrient uptake

Plants combined with AMF can facilitate growth and nutrient accumulation (Wicaksono et al., 2018; Real-Santillán et al., 2019). The AMF significantly increased the seedling height, ground diameter, biomass, N accumulation and P accumulation (**Figures 2A–C**; **Figures 3A, B**). Generally, AMF colonizing the host plant’s root system can improve plant growth and nutrient accumulation by extending the root absorption area (Marschner and Dell, 1994; Bourles et al., 2020). In particular, the biomass and nutrient accumulation increased with increasing gravel content when AMF colonized *B. pilosa* (**Figure 2C**; **Figures 3A, B**). A previous study showed that higher gravel content could increase soil porosity and allow a higher oxygen supply, thus supporting higher mycorrhizal colonization (Fu et al., 2022), which was consistent with the root mycorrhizal colonization rate showed that HG > LG > FG (**Table 1**). Thus, the substrate may affect plant growth and nutrient uptake by influencing root mycorrhizal colonization, which explains the interaction of AM × SG significantly influenced the growth and nutrition of *B. pilosa* (**Table 2**, **3**). Additionally, the extraradical mycelium is thinner than the plant root system, enabling it to obtain nutrients across narrow soil porosity easily, thus improving plant biomass and nutrient accumulation (Hawkes et al., 2011; Martin et al., 2012; Jones and French, 2021). Therefore, these results showed that AMF increased the growth and nutrition of plants more in the substrate mixed with gravel than in the free gravel substrate in heterogeneous karst habitats, which is consistent with H1.

In this study, AMF induced higher growth and nutrition in small patches than in large patches (**Figures 2A–C**; **Figures 3A, B**), which is inconsistent with H2. On the one hand, when resources are spatially patchy, plants develop a foraging response in which plants selectively place resource acquisition organs in favorable patches of heterogeneous environments (Hutchings and de Kroon, 1994; Roiloa and Retuerto, 2006; Dong et al., 2015). Therefore, the plant foraging is unrestricted and coarse-grained under the large patch with the free gravel substrate in heterogeneous conditions (Wijesinghe and Hutchings, 1997). However, under the heterogeneous small patch with the free gravel substrate, the restriction of gravel in the adjacent heterogeneous small patch (**Figure 1C**) hinders plant roots from obtaining nutrients from adjacent and even more distant free gravel patches, thus inhibiting plant growth and nutrient accumulation (Kume et al., 2006). These results explain that plant growth and nutrition were higher in Hetl than in Hets with M^-^ treatment, which may be due to the plant root system being more restricted in heterogeneous small patches than in heterogeneous large patches. On the other hand, with M^+^ treatment, the root system of *B. pilosa* under the heterogeneous small patch with free gravel substrate expanded the nutritional area in soil touched by the root system due to the joining of mycelium. The mycelium could acquire nutrients in adjacent patches and more distant patches without being hindered by gravel (Adeyemi et al., 2021; Yan et al., 2021), thus allowing the *B. pilosa* to grow better under the heterogeneous small patch than under the heterogeneous large patch with the free gravel substrate. These results can be demonstrated in **Table 1**, which shows that the root mycorrhizal colonization rate under Hets with FG substrate was significantly higher than under Hetl with FG substrate. Therefore, the plant growth and nutrition were higher in Hets than in Hetl through AM mycelium, which explains that the interaction of AM × SP significantly influenced the growth and nutrition of *B. pilosa* (**Tables 2**, **3**). In addition, the plant growth and nutrient uptake under heterogeneous patches with high gravel content were higher than under homogeneous patches (**Figures 2A–C**; **Figures 3A, B**). Previous studies suggested that mycorrhizal symbiosis could promote plant preemption for limited resources when soil nutrients are heterogeneous (Facelli and Facelli, 2002). Therefore, plants under heterogeneous patch conditions with high gravel content may preempt heterogeneous nutrient resources through mycorrhizal symbiosis, resulting in higher plant growth and nutrient accumulation than under homogeneous patch conditions (Hutchings and John, 2004; Croft et al., 2012).

Many plants adjust the R/S ratio to respond to resource imbalances (Reynolds and D'antonio, 1996; Aphalo et al., 1999; Song et al., 2016). In this study, the R/S ratio in HG substrate was higher than in FG substrate under M^-^ treatment (**Figure 2D**). The gravel content in soil significantly affects plant growth by increasing the difficulty of plant roots in obtaining nutrients and decreasing the soil nutrient availability (Ercoli et al., 2006; Mi et al., 2016). Fu et al. (2022) also demonstrated that large amounts of gravel could reduce the availability of nutrients, and the root system tends to exhibit a slow turnover of fine roots. Therefore, in high gravel content soils, *B. pilosa* allocated more resources to the belowground to establish a root system to acquire nutrients, which led to a larger R/S ratio than in the soil with the free gravel substrate. These results were consistent with the OPT (Bloom et al., 1985), i.e., plants prefer to allocate resources to the root system in the limited resources when soil nutrient availability is low. AMF can regulate the resource allocation of host plants between aboveground and belowground (Nouri et al., 2015; He et al., 2019). In this study, contrary to M^-^ treatment, the R/S ratio in FG substrate was higher than in HG substrate under M^+^ treatment (**Figure 2D**). It is more carbon-expensive for plants to build fine roots than to invest in thin fungal mycelial extensions (Hodge, 2004). Therefore, when inoculated with AMF under the high gravel content substrate, *B. pilosa* might allocate more resources to the aboveground part for obtaining more photosynthetic products and exchanging the required nutrients with AMF (Stock et al., 2021). It leads to the mycelium partially replacing the function of the root system (Lanfranco et al., 2018), as evidenced by the root mycorrhizal colonization rate in the high gravel substrate being higher than in the free gravel substrate (**Table 1**). Therefore, these results potentially demonstrate that the cooperative relationship between plants and AMF in heterogeneous habitats may result in a deviation of biomass allocation from the OPT spectrum.

### Plant growth and nutrition associated with mycorrhizae depend mainly on the gravel content rather than the patch size

Previous studies have concluded that plant growth and nutrient uptake depend on the heterogenous scale, although under the same amount of substrate conditions (Wijesinghe and Hutchings, 1997; van der Waal et al., 2011; Wang et al., 2016). However, in our study, the effect of gravel content, rather than patch size, on the LnRR for plant growth and nutrient accumulation of *B. pilosa* was significant (**Figure 4**), which is inconsistent with H3. One possible reason is that plants adopt effective measures to cope with the heterogeneous resource environment (Wang et al., 2017), particularly in karst habitats. This explanation is supported by the fact that the spatial heterogeneity of the karst habitat is mainly influenced by gravel content (Zhang et al., 2018). Additionally, a strong negative correlation was found between gravel content and resource availability in soil (Fu et al., 2022). Thus, the substrate gravel may significantly influence the plant growth and nutrient accumulation of *B. pilosa* by affecting soil nutrient availability in this study. Further, in soils where resources are scarcer, plants depend more on mycelium, which allows them to access resources from farther away patches (Wang et al., 2016; Gomes et al., 2019). It reduces the sensitivity of plant growth and nutrient uptake to patch size. Therefore, in this study, the LnRR in growth and nutrients of the substrate gravel was significantly higher than the substrate patchiness when inoculated with AMF. It indicated that plant growth and nutrient accumulation associated with AMF mainly depend on the gravel content rather than the patch size in the heterogeneous karst habitat. These findings may provide a theoretical reference for vegetation restoration in highly heterogeneous karst ecosystems.

## Conclusions

In this study, AMF significantly increased the growth and nutrition of karst plant *B. pilosa* under substrate patchiness and substrate gravel treatments. The substrate mixed with gravel promoted higher growth and nutrients of *B. pilosa* than the free gravel substrate when inoculated with AMF. The heterogeneous small patch enhanced the growth and nutrients of *B. pilosa* more than the heterogeneous large patch when inoculated with AMF. By analyzing the response ratio LnRR, AMF was involved in higher growth and nutrition of *B. pilosa* under the substrate gravel than under the substrate patchiness. We concluded that the plant growth and nutrition regulated by AMF depend mainly on the substrate gravel content rather than the spatial patchiness in the heterogeneous karst habitat.

## Data availability statement

The raw data supporting the conclusions of this article will be made available by the authors, without undue reservation.

## Author contributions

YH designed the experiments. XX and HX conducted the experiments, and KS wrote the first draft of the manuscript. KS, TX, LG, MJ, XY, and JY analyzed the data. KS, YH, XL, YG, BW, and LZ revised and proofread the manuscript, and MU edited the language. All authors contributed to the article and approved the submitted version.

## Funding

This study was supported by the National Natural Science Foundation of China (NSFC: 32260268), the Science and Technology Project of Guizhou Province [(2021) General-455], the Guizhou Hundred-level Innovative Talents Project [Qian-ke-he platform talents (2020) 6004].

## Conflict of interest

The authors declare that the research was conducted in the absence of any commercial or financial relationships that could be construed as a potential conflict of interest.

## Publisher’s note

All claims expressed in this article are solely those of the authors and do not necessarily represent those of their affiliated organizations, or those of the publisher, the editors and the reviewers. Any product that may be evaluated in this article, or claim that may be made by its manufacturer, is not guaranteed or endorsed by the publisher.
